# Gene Co-expression Network and Copy Number Variation Analyses Identify Transcription Factors Associated With Multiple Myeloma Progression

**DOI:** 10.3389/fgene.2019.00468

**Published:** 2019-05-17

**Authors:** Christina Y. Yu, Shunian Xiang, Zhi Huang, Travis S. Johnson, Xiaohui Zhan, Zhi Han, Mohammad Abu Zaid, Kun Huang

**Affiliations:** ^1^Department of Biomedical Informatics, The Ohio State University, Columbus, OH, United States; ^2^Department of Medicine, Indiana University School of Medicine, Indianapolis, IN, United States; ^3^Department of Medical and Molecular Genetics, Indiana University, Indianapolis, IN, United States; ^4^National-Regional Key Technology Engineering Laboratory for Medical Ultrasound, Guangdong Key Laboratory for Biomedical Measurements and Ultrasound Imaging, School of Biomedical Engineering, Health Science Center, Shenzhen University, Shenzhen, China; ^5^School of Electrical and Computer Engineering, Purdue University, West Lafayette, IN, United States; ^6^Regenstrief Institute, Indianapolis, IN, United States

**Keywords:** multiple myeloma, MGUS, SMM, gene co-expression, copy number variation

## Abstract

Multiple myeloma (MM) has two clinical precursor stages of disease: monoclonal gammopathy of undetermined significance (MGUS) and smoldering multiple myeloma (SMM). However, the mechanism of progression is not well understood. Because gene co-expression network analysis is a well-known method for discovering new gene functions and regulatory relationships, we utilized this framework to conduct differential co-expression analysis to identify interesting transcription factors (TFs) in two publicly available datasets. We then used copy number variation (CNV) data from a third public dataset to validate these TFs. First, we identified co-expressed gene modules in two publicly available datasets each containing three conditions: normal, MGUS, and SMM. These modules were assessed for condition-specific gene expression, and then enrichment analysis was conducted on condition-specific modules to identify their biological function and upstream TFs. TFs were assessed for differential gene expression between normal and MM precursors, then validated with CNV analysis to identify candidate genes. Functional enrichment analysis reaffirmed known functional categories in MM pathology, the main one relating to immune function. Enrichment analysis revealed a handful of differentially expressed TFs between normal and either MGUS or SMM in gene expression and/or CNV. Overall, we identified four genes of interest (*MAX*, *TCF4*, *ZNF148*, and *ZNF281*) that aid in our understanding of MM initiation and progression.

## Introduction

Multiple myeloma (MM) is a B-cell malignancy caused by the proliferation of aberrant clonal plasma cells that secrete monoclonal immunoglobulin protein, also known as M protein. MM is consistently preceded by a premalignant phase called monoclonal gammopathy of undetermined significance (MGUS) and clinically defined by thresholds in serum M protein and clonal bone marrow plasma cell content with the absence of hypercalcemia, renal insufficiency, anemia, and bone lesions (known as CRAB features) or amyloidosis relating to the plasma cell proliferative disorder (Landgren et al., 2009; Rajkumar et al., 2014). The risk of developing MGUS is low, thought to be around 3.2% of individuals aged 50 or older and increases to 5.3% for those aged 70 or older (Kyle et al., 2006). An individual with MGUS lives with an increased risk of developing MM at a rate of 1% per year (Kyle et al., 2002). Additionally, there is an intermediate precursor between MGUS and MM known as smoldering multiple myeloma (SMM). This phase is clinically defined by a higher threshold in M-protein or clonal bone marrow plasma cell content with the continued absence of CRAB features (Rajkumar et al., 2014). The risk of progression for SMM increases at a variable rate, as 10% per year for the first 5 years, 3% per year for the next 5 years, and 1% per year in the following 10 years (Kyle et al., 2007). Understanding the biological basis of MM progression from these precursors is still unclear.

Gene expression profiling studies have been applied to MM to identify subgroups and biomarkers in order to better understand the molecular basis of disease, improve prognostic models, and characterize features associated with a high risk of disease progression (Davies et al., 2003; Zhan et al., 2006; Chng et al., 2007a; Shaughnessy et al., 2007; Broyl et al., 2010; Dhodapkar et al., 2014; López-Corral et al., 2014; Shao et al., 2018). A few studies have analyzed the disease precursors using hierarchical clustering and differential expression analysis to identify gene signatures (Davies et al., 2003; Zhan et al., 2007; López-Corral et al., 2014). We approached gene expression profiling analysis from the transcription factor (TF) perspective, using gene co-expression networks (GCNs).

Gene co-expression networks have been widely used in discovery of new gene functions and regulatory relationships (Langfelder and Horvath, 2008; Zhang et al., 2010, 2012; Kais et al., 2011; Yin et al., 2015; Zhang and Huang, 2016; Miao et al., 2018). GCNs have been implemented in a few MM studies albeit these studies focused on differential gene expression and not co-expression (Dong et al., 2015; Wang et al., 2016; Liu et al., 2017). We applied GCN analysis on two publicly available MM datasets to identify regulatory genes specifically associated with or disrupted in MM precursors.

The GCN algorithm we employed is local maximal Quasi-Clique Merger (lmQCM) (Zhang and Huang, 2016), previously developed to mine densely correlated gene modules in weighted GCNs (Zhang et al., 2010; Zhang and Huang, 2016, 2017; Xiang et al., 2018). The advantages that lmQCM has over a similar method such as WGCNA (Langfelder and Horvath, 2008) is the ability to allow genes to belong to more than one module and the ability to produce smaller sized modules many of which are related to copy number variations (CNVs) in cancers (Han et al., 2016; Zhang and Huang, 2016; Xiang et al., 2018).

We further supported and validated our gene expression findings with CNVs from microarray technology based on single-nucleotide polymorphism (SNP) arrays. SNP arrays can be used in numerous ways to identify genomic imbalances (She et al., 2008; López-Corral et al., 2012; Johnson et al., 2016; Mitchell et al., 2016; Mikulasova et al., 2017). We surmised that some gene expression changes from normal to MM precursors can be explained by CNVs in order to better understand the genomic changes of myeloma progression.

## Materials and Methods

### Gene Expression Profiling Datasets: Processing and GCN

We applied an integrative network-based approach to identify modules of co-expressed genes associated with MM precursors. MM microarray datasets GSE5900 and GSE6477 from the Gene Expression Omnibus (GEO) were obtained, annotated, and filtered using the TSUNAMI web-tool^1^. The web-tool retrieved the gene expression matrices via the R package GEOquery. We converted probe IDs to corresponding HGNC symbols according to GEO Platform accession number. In the case of duplicate gene symbols, we retained the one with the largest mean expression value. Probes without gene symbols were removed. We further filtered the data by removing the lowest 20% of genes quantified by absolute average value. The lowest 50% of genes quantified by variance in GSE5900 were removed, while filtering GSE6477 was accomplished by removing the lowest 10% of genes quantified by absolute average value and lowest 10% of genes quantified by variance. We applied a stricter cutoff on GSE5900 because the microarray platform had a much larger probeset than the platform in GSE6477 (54,675 vs. 22,283 probes). This was conducted in order to obtain expression sets with similar numbers of genes. The resulting datasets had 15,388 and 12,530 genes for GSE5900 and GSE6477, respectively. Normalization of the datasets was confirmed by inspecting the boxplots of the samples for consistent median values.

### SNP Array Dataset: Processing and CNV Analysis

We obtained raw CEL files from GEO study GSE31339, sequenced on Affymetrix Genome-Wide Human SNP Array 6.0. The CEL files were analyzed by the R package Rawcopy (Mayrhofer et al., 2016) and then aggregated by the following conditions: normal (*n* = 10), MGUS (*n* = 20), and SMM (*n* = 19). SMM sample GSM777173 was removed from our analysis after the sample identity distogram suggested some cell or DNA contamination with other samples (Supplementary Figure S1). CNVs were detected in genomic segments using PSCBS, an enhanced method of circular binary segmentation (Bengtsson et al., 2010; Olshen et al., 2011). We used the reference data included in Rawcopy for calculating logarithm (base 2) ratios (log_2_ ratios) of genome segmentation. Rawcopy defined the thresholds for copy number gain as segment median log_2_ ratio > 0.2 and copy number losses as segment median log_2_ ratio < −0.3 (Mayrhofer et al., 2016). The package also annotated probes with their corresponding genes.

### Gene Co-expression Network Mining

We separated GSE5900 into three datasets: normal (*n* = 22), MGUS (*n* = 44), and SMM (*n* = 12). The GSE6477 dataset was separated in the same fashion into three datasets: normal (*n* = 15), MGUS (*n* = 22), and SMM (*n* = 22). GCN mining was conducted using the R package lmQCM. The lmQCM algorithm has an option for normalizing the edge weights of the weighted co-expression network by setting the sums of both rows and columns of the weight matrix to be all ones similar to the weight normalization in spectral clustering (Ng et al., 2001). Another important parameter for lmQCM is gamma that controls the initiation of new gene modules in the iterative mining process. Here, we applied the edge weight normalization and also tested varying gamma values; the rest of the parameters were kept as the default. The normalization process suppresses high weights between nodes and boosts edges with relatively lower weights, which overcomes the issue of unbalanced edge weights in dense module mining algorithms (Zhang and Huang, 2016). The gamma variable ranges from 0 to 1 and controls for the number of generated modules and the maximum module size. For normalized weights, the suggested range of gamma is 0.3–0.75. A higher gamma results in more total modules with fewer genes in the largest module. A lower gamma results in less total modules with more genes in the largest module. We selected gamma values that struck a balance between these two outcomes and elected to keep the largest module under 500 genes. Different values for gamma were selected to obtain a similar number of modules between the same conditions (i.e., normal, MGUS, or SMM) in GSE5900 and GSE6477. This allows the identified modules to be more comparable between datasets of the same condition. We chose the following gamma values for GSE5900: 0.60 for normal, 0.40 for MGUS, and 0.75 for SMM. The following gamma values were chosen for GSE6477– normal: 0.65, MGUS: 0.60, and SMM: 0.55.

For comparison, we also applied the widely used weighted GCN mining algorithm WGCNA (Langfelder and Horvath, 2008) on the same datasets specifying a minimum module size of 10 and using power 5 or 6 as appropriate, leaving the rest of the settings as default. We then selected the most similar modules from lmQCM and WGCNA and calculated gene-wise Spearman correlations to quantify the co-expression density of each module. The most similar modules were determined using the Jaccard index between lmQCM and WGCNA modules in the same condition, where the Jaccard index is simply defined as the size of the intersection between two gene modules divided by the size of the union of the same two modules.

### Identification of Condition-Specific Modules

Condition-specific modules are those in which the expression profile of the genes in one module is more correlated in one condition compared to others (e.g., normal, MGUS, or SMM). We utilized a previously developed metric called Centralized Concordance Index (CCI) that evaluates the co-expression of genes within modules identified from GCN analysis (Han et al., 2016). The CCI describes how strongly genes co-express and is calculated from a subset of gene expression data containing the genes from a module and samples from a single condition. CCI values range from 0 to 1, with a higher number indicating more densely correlated genes. For each gene module identified from lmQCM, we calculated the corresponding CCI in normal, MGUS, and SMM. The CCIs for each module were then compared across the three conditions, and a difference of ∼0.2 in CCI values between MM precursors (MGUS or SMM) and normal were identified as potentially interesting.

### Module Similarity Between Datasets

We further reduced our modules of interest by identifying modules with similar genes between GSE5900 and GSE6477. The Jaccard index, described above in Section “Gene Co-expression Network Mining,” was used to calculate the similarity of modules in the same conditions between GSE5900 and GSE6477. This calculation was conducted between every pair of modules in each condition: normal, MGUS, and SMM. Each resulting matrix was then transformed into a *z*-score where the top one percentile of similar module pairs from each condition were kept to filter the list of potentially interesting modules for enrichment analysis.

### Functional Enrichment Analysis and Identification of Upstream Regulators

We used the R package enrichR (Kuleshov et al., 2016) to conduct enrichment analysis of the genes in each module of interest. We specified the “GO Biological Process 2017b” and “KEGG 2016” databases for functional and pathway enrichment analyses. For determining the significance of GO and KEGG pathway terms, we used Bonferroni significance cutoffs of 0.05/nMods where nMods is the number of modules corresponding to the specific dataset. For instance, the *p*-value cutoff for GSE5900 normal-specific data is 0.05/31 = 0.00161. We took GO terms with significant *p*-values and summarized them using the web-tool REVIGO (Supek et al., 2011).

Using enrichR, we specified the “TRANSFAC and JASPAR PWMs” database to identify TFs that regulate the genes in our modules of interest, using a less stringent Bonferroni cutoff of 0.1/nMods. We then narrowed down the list of TFs by identifying those that were differentially expressed among the three conditions by either gene expression data or CNV segment median data by conducting Mann–Whitney tests between normal and MGUS and between normal and SMM samples.

### Network Analysis of TF Targets

We used Ingenuity Pathways Analysis (IPA, Qiagen) for network analysis of TFs and their targets determined from enrichR to explore possible signaling pathways. We conducted *core analyses* (which is a function of IPA) for each TF and its targets, using experimentally observed knowledge in the Ingenuity Knowledge Base and specifying direct and indirect gene relationships in human tissue and cell lines.

## Results

### lmQCM Produces Smaller-Sized Modules Than WGCNA With Stronger Gene Correlations

**FIGURE 1:**
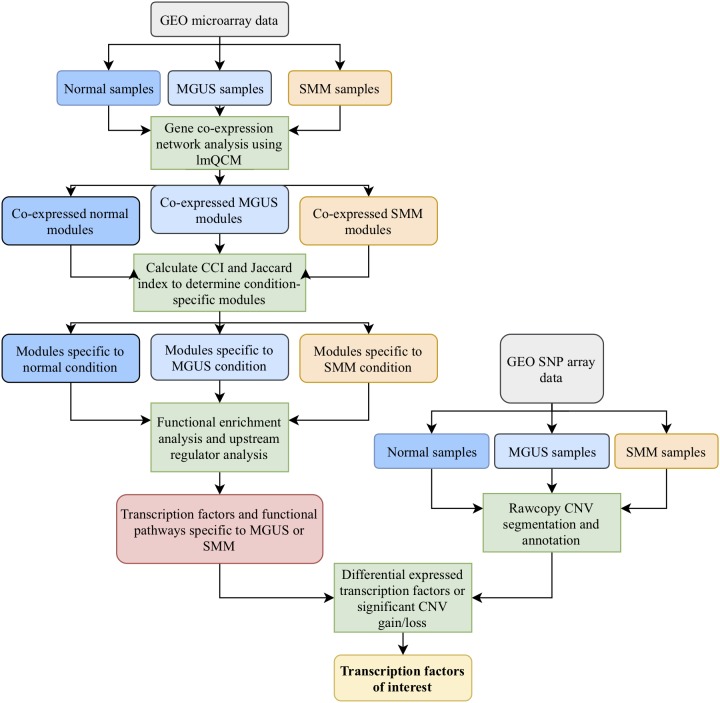
Workflow of the methods employed in this study.

Our workflow is shown in Figure 1. After applying the lmQCM algorithm using the specified gamma values to the GSE5900 datasets, we obtained 78, 60, and 95 modules for normal, MGUS, and SMM, respectively; module sizes ranged from 10 to 400 genes. In GSE6477, using the specified gamma values, we obtained 79, 85, and 70 modules for the normal, MGUS, and SMM samples, respectively; module sizes ranged from 10 to 352 genes. Applying WGCNA to GSE5900, we obtained 40, 41, and 98 modules for normal, MGUS, and SMM, respectively; module sizes ranged from 11 to 4694 genes. In applying WGCNA to GSE6477, we obtained 34, 99, and 74 modules for normal, MGUS, and SMM, respectively; module sizes ranged from 11 to 4324 genes. Detailed breakdowns by sample type are shown in Table 1.

**Table 1:** GCN results from algorithms lmQCM and WGCNA.

Dataset	Sample type	Sample size	lmQCM total modules	lmQCM module sizes	WGCNA total modules	WGCNA module sizes
GSE5900	Normal	22	78	10–400	40	12–1943
GSE5900	MGUS	12	60	10–332	41	12–4694
GSE5900	SMM	44	95	10–236	98	11–2732
GSE6477	Normal	15	79	10–119	34	11–4324
GSE6477	MGUS	22	85	10–352	99	13–1494
GSE6477	SMM	24	70	10–248	74	11–1652

*The total number of resulting modules and size range are detailed by dataset and sample type.*

The most similar gene modules were identified from two SMM modules in lmQCM and WGCNA. The lmQCM module contained 224 genes and the WGCNA module contained 393 genes. The Jaccard index was 0.396, with an overlap of 175 genes. Within each respective module, we calculated the Spearman correlation in a gene-wise manner and conducted a two-sided Mann–Whitney test between the absolute value of the correlation coefficients in each population. The correlation coefficients were significantly higher in the lmQCM module (median: 0.399) compared to the WGCNA module (median: 0.322) with a *p*-value of 2.2E-16 (Supplementary Figure S2).

### Module Reduction Using CCI and Jaccard Similarity

Normal-, MGUS-, and SMM-specific modules were identified by calculating the CCI difference between normal and MGUS samples and normal and SMM samples and setting a cutoff of around 0.2 CCI difference. This resulted in 68 and 79 normal-specific modules, 45 and 72 MGUS-specific modules, 95 and 63 SMM-specific modules across GSE5900 and GSE6477 datasets, respectively. An example of a normal-specific gene module is visualized using Spearman correlation heatmaps in Supplementary Figure S3.

To further reduce modules of interest, we used Jaccard similarity. After module similarity comparison using the Jaccard index, we reduced the interesting modules to more manageable numbers than solely using CCI and were left with 31 and 39 normal-specific modules, 22 and 31 MGUS-specific modules, and 47 and 30 SMM-specific modules across GSE5900 and GSE6477 datasets, respectively. The module sizes ranged from 10 to 400 genes.

### Frequency of CNVs Increase From MGUS to SMM

**FIGURE 2:**
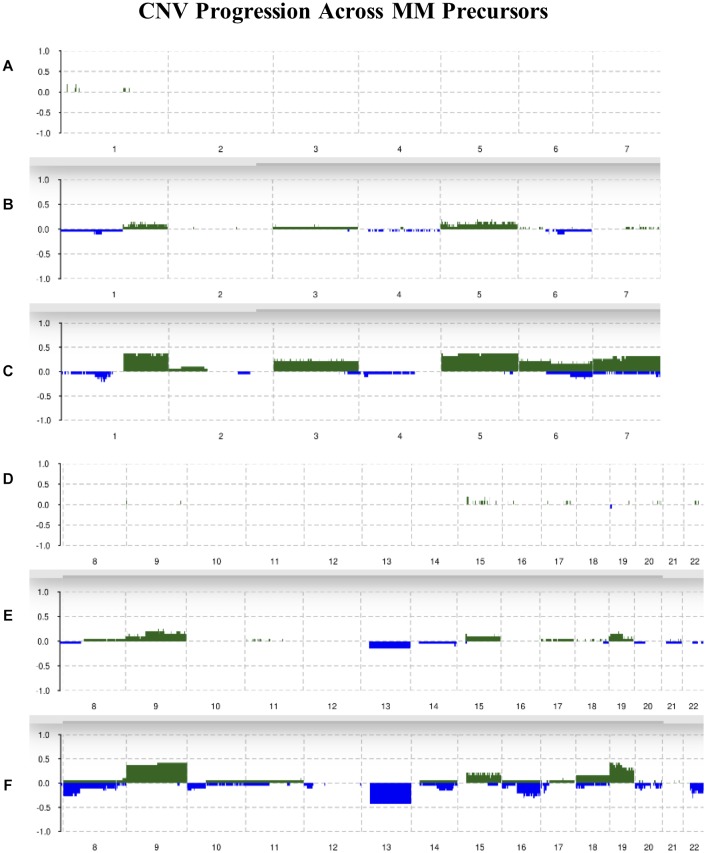
Summary of CNVs across the genome from chromosomes 1–7 in **(A)** normal, **(B)** MGUS, and **(C)** SMM samples. Summary of CNVs across the genome from chromosomes 8–22 in **(D)** normal, **(E)** MGUS, and **(F)** SMM samples. The *y*-axis indicates the frequency of the chromosomal aberration. Green indicates amplification; blue indicates deletion.

Chromosomes 2, 4, 10, 11, 12, and 21 were mostly unchanged and showed 10% or less allelic imbalance in all conditions. Chromosomes 1q, 3, 5, 6, 7, 9, 15, 18, and 19 were slightly amplified in MGUS and more amplified in SMM, with chromosomes 1q, 5, 9, and 19 showing the highest frequencies of change in SMM of around 40%. For instance, 1q had about 10% of MGUS samples amplified and around 40% of SMM samples amplified. We observed an increased frequency of deletions in chromosomes 1p, 6, 7, 8p, 10, 12p, 13, 14q, 16q, 18, 20, and 22q; the highest deletion frequency was around 25% and was observed in 8p, 13, 16q, and 22q of SMM patients. The CNV landscape across conditions is shown in Figure 2.

### EnrichR GO Results Are Highly Enriched in Immune-Related Terms

The top GO BP terms from all condition-specific modules are shown in Supplementary Table S1. In the normal-specific data, there were 95 significant GO BP terms that appeared in both GSE5900 and GSE6477, the top few being neutrophil degranulation, antigen processing and presentation of exogenous peptide antigen via MHC class II, and antifungal humoral response. These GO terms are mostly related to immune system response.

The MGUS-specific data had 40 significant GO BP terms in common from GSE5900 and GSE6477, with many immune function terms such as positive regulation of B cell activation, response to interferon-alpha, and B cell receptor signaling pathway.

The SMM-specific data shared 125 GO BP terms between GSE5900 and GSE6477 data, the most significant ones relating to the process of transcription and translation. There were also terms related to immune function such as B cell receptor signaling pathway.

### Condition-Specific Modules From Four Identified TFs Describe Different Aspects of Myeloma

We identified these TFs as interesting: *MAX*, *TCF4*, *ZNF148*, and *ZNF281*. *MAX* was identified from a normal-specific module, *TCF4* and *ZNF148* were identified from MGUS-specific modules, and *ZNF281* was identified from a SMM-specific module. Three TFs (*MAX*, *TCF4*, and *ZNF148*) were differentially expressed between normal and a MM precursor (MGUS or SMM) in the gene expression datasets and/or the CNV dataset (Table 2). While *ZNF281* was not differentially expressed, it showed an interesting increase in copy number gain from normal to MGUS and to SMM.

**Table 2:** Transcription factors of interest, identified from condition-specific modules in normal, MGUS, and SMM samples.

Transcription factor	Chromosomal region	TF targets
*MAX*	14q12-q24	*NLGN4X*, *VEGFB*, *STMN3*, *CTSW*, *OVOL1*, *SGSH*, *PDP1*, *LYL1*, *DRAM1*, *SH3BP1*, *ZMIZ1*, *NFIC*, *RGL3*, *PTPRCAP*, *FGF13*, *CUEDC1*
*ZNF148*	3q13-q22	*NLGN4X*, *VEGFB*, *STMN3*, *CTSW*, *OVOL1*, *SGSH*, *PDP1*, *LYL1*, *DRAM1*, *SH3BP1*, *ZMIZ1*, *NFIC*, *RGL3*, *PTPRCAP*, *FGF13*, *CUEDC1*
*TCF4*	18q11-q23	*UEVLD*, *DSP*, *ALS2CR11*, *NT5E*, *RALYL*, *EFEMP1*, *GEMIN5*, *PPARGC1A*
*ZNF281*	1q32-q44	*HRK*, *SLC26A1*, *TNXB*, *CRABP2*, *IBA57*, *LOC728392*, *ESPN*, *AGPAT2*, *HS6ST1*, *DLL3*, *IL4I1*, *RGS3*, *FUT7*, *PDLIM2*, *NUP62*, *POLR2F*, *GGT1*, *SLC38A3*, *ZBTB7B*, *POLR2J*, *WNT2*, *MUC6*, *POLR2J3*, *WWTR1*, *PDIA2*, *KLF12*, *ZFHX3*, *ACE*, *POLR2J2*, *SLC2A11*, *GP1BB*, *ABCA3*, *XRCC1*, *FNDC11*, *CTAG2*, *RENBP*, *CLDN5*, *DLG4*, *TRPV4*, *NOX5*, *IGFALS*, *HOXB8*

*The chromosomal regions were determined by Rawcopy. The TF targets were identified by enrichR.*

#### Module Descriptions

The gene co-expression module containing *MAX* was functionally enriched in bleb assembly and activation of MAPKKK activity involved in innate immune response.

The gene co-expression module containing *ZNF148* was functionally enriched in antigen processing and presentation of exogenous peptide antigen via MHC class II and negative regulation of peptide hormone processing.

In the gene co-expression module containing *TCF4*, multiple assembly complexes containing the genes *GEMIN5*, *PPARGC1A*, and *TEAD1* were significantly enriched. They include apoptosome assembly, mitotic checkpoint complex assembly, and Wnt signalosome assembly.

The gene co-expression module containing *ZNF281* is functionally enriched in genes involved in transcription. These include transcription, DNA-templated, transcription from RNA polymerase II promoter, telomeric repeat-containing RNA transcription, and mRNA transcription.

The details of GO BP enrichment results (top enriched terms and *p*-values) for these modules with their corresponding *p*-values are listed in Supplementary Table S1.

#### TFs Exhibit Consistent CNV and Gene Expression Trends During the Course of Myeloma Progression

**FIGURE 3:**
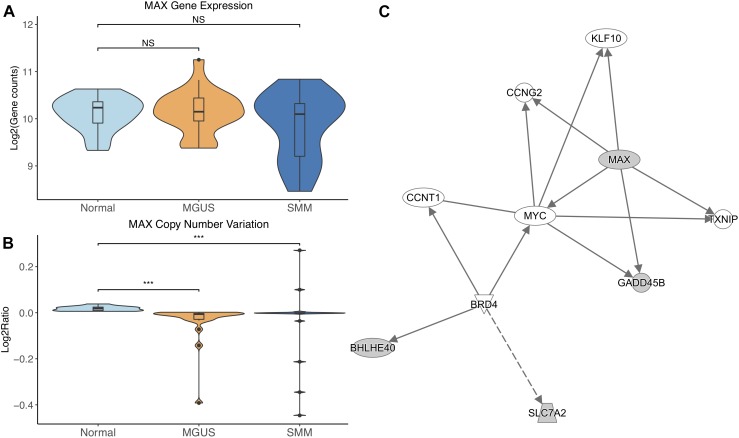
**(A)** *MAX* expression across sample groups. Mann–Whitney tests between groups showed no significant difference. **(B)** Observations of *MAX* copy number. Mann–Whitney tests showed significant copy number variation between Normal and MGUS (*p* = 1.17E-05) and between Normal and SMM (*p* = 6.10E-04). **(C)** A predicted interaction network of *MAX* and its downstream targets. The gray nodes indicate genes from our module and the white nodes are gene interactions defined in IPA. Solid lines between nodes indicate a direct interaction supported by the Ingenuity Knowledge Base while the dashed line indicates an indirect interaction. Significance levels: ^∗^*p* ≤ 0.05; ^∗∗^*p* ≤ 0.01; ^∗∗∗^*p* ≤ 0.001.

*MAX* did not show differential gene expression; however, its copy number significantly decreased in MGUS and SMM compared to normal (*p*-val = 1.17E-05 and 6.10E-04, respectively, Figure 3A,B). The CNV pattern showed deletions in MGUS and amplification and deletions in SMM (Figure 3B).

**FIGURE 4:**
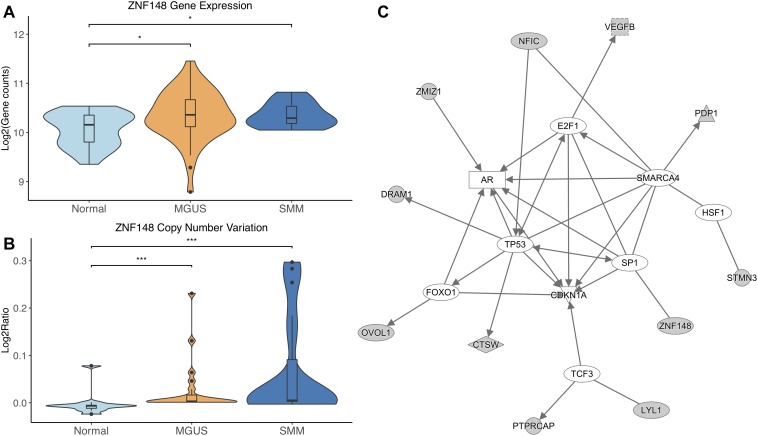
**(A)** *ZNF148* expression across sample groups. Mann–Whitney tests showed significant differential expression between Normal and MGUS (*p* = 1.75E-02) and between Normal and SMM (*p* = 4.05E-02). **(B)** Observations of *ZNF148* copy number. Mann–Whitney tests showed significant copy number variation between Normal and MGUS (*p* = 4.76E-04) and between Normal and SMM (*p* = 3.11E-04). **(C)** A predicted interaction network of *ZNF148* and its downstream targets. The gray nodes indicate genes from our module and the white nodes are gene interactions defined in IPA. Solid lines between nodes indicate a direct interaction supported by the Ingenuity Knowledge Base. Significance levels: ^∗^*p* ≤ 0.05; ^∗∗^*p* ≤ 0.01; ^∗∗∗^*p* ≤ 0.001.

*ZNF148* was the only TF that showed significantly different CNV aberrations and gene expression, with gene expression and copy number amplification both increasing in MGUS and SMM (*p*-val range: 1.75E-02–3.11E-04, Figure 4A,B).

**FIGURE 5:**
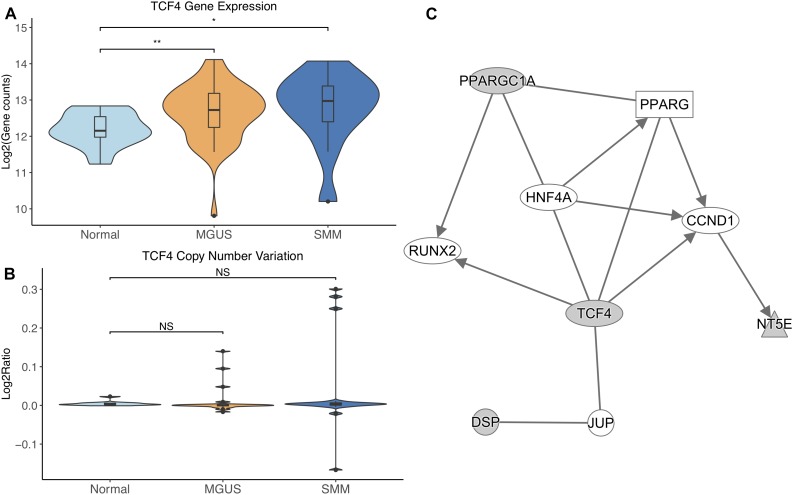
**(A)** *TCF4* expression across sample groups. Mann–Whitney tests showed significant differential expression between Normal and MGUS (*p* = 3.65E-03) and between Normal and SMM (*p* = 1.49E-02). **(B)** Observations of *TCF4* copy number. Mann–Whitney tests showed no significant differences between any groups. **(C)** A predicted interaction network of *TCF4* and its downstream targets. The gray nodes indicate genes from our module and the white nodes are gene interactions defined in IPA. Solid lines between nodes indicate a direct interaction supported by the Ingenuity Knowledge Base. Significance levels: ^∗^*p* ≤ 0.05; ^∗∗^*p* ≤ 0.01; ^∗∗∗^*p* ≤ 0.001.

*TCF4* was differentially expressed between normal/MGUS (*p*-val = 3.65E-03) and normal/SMM (*p*-val = 1.49E-02), with gene expression progressively increasing from MGUS to SMM (Figure 5A). In regard to CNVs, *TCF4* exhibited amplifications in MGUS and amplifications and deletions in SMM (Figure 5B).

**FIGURE 6:**
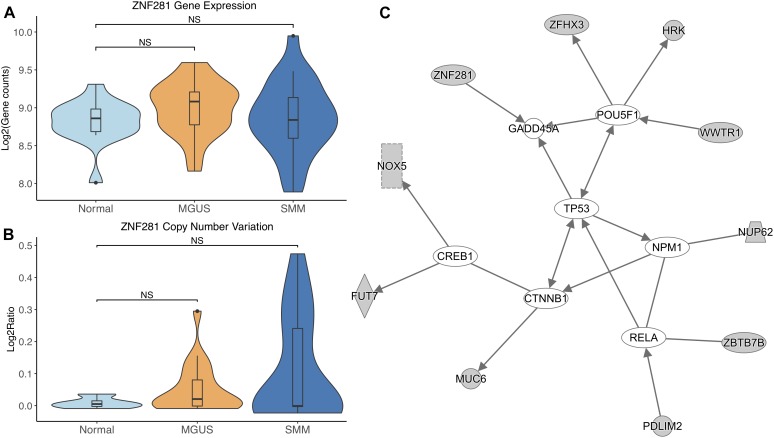
**(A)** *ZNF281* expression across sample groups. Mann–Whitney tests between groups showed no significant difference. **(B)** Observations of *ZNF281* copy number. Mann–Whitney tests showed no significant differences between any groups. **(C)** A predicted interaction network of *ZNF281* and its downstream targets. The gray nodes indicate genes from our module and the white nodes are gene interactions defined in IPA. Solid lines between nodes indicate a direct interaction supported by the Ingenuity Knowledge Base. Significance levels: ^∗^*p* ≤ 0.05; ^∗∗^*p* ≤ 0.01; ^∗∗∗^*p* ≤ 0.001.

*ZNF281* did not show differential gene expression (Figure 6A). *ZNF281* showed increasing CNV amplifications from MGUS to SMM, but it was not considered significant by Mann–Whitney tests (Figure 6B).

#### TF Signaling Networks Are Related to Cancer Progression

IPA network analysis showed *MAX* and its targets interact with other TFs *CCNT1*, *KLF10*, and *MYC*. *MAX* is further predicted to target *CCNG2* and *TXNIP*. *BRD4* is shown to regulate expression of *BHLHE40* and *SLC7A2* (Figure 3C).

*ZNF148* and its targets were shown to interact with TFs *TP53*, *FOXO1*, *SP1*, *TCF3*, *HSF1*, *SMARCA4*, and *E2F1*. Additionally, *CDKN1A* was shown to be a common target of the TFs listed above (Figure 4C).

*TCF4* and its targets were shown to interact with TFs *RUNX2*, *CCND1*, and *HNF4A* in addition to nuclear receptor *PPARG* and junction protein *JUP* (Figure 5C).

*ZNF281* and its targets were shown to interact with TFs *CREB1*, *CTNNB1*, *RELA*, *NPM1*, and *POU5F1*. *ZNF281* was shown to directly target *GADD45A*. *TP53* was shown to be an intermediate interactor that connected each subnetwork (Figure 6C).

## Discussion

We conducted GCN analyses on two publicly available MM datasets and identified four TFs by a condition-specific method. This pipeline has previously not been applied to studying MM precursors. Our approach identified TFs expressed in condition-specific gene modules in publicly available MM data. We then validated our TFs with CNV data taken from a third publicly available dataset, looking for genes located on chromosomal segments that showed a consistent trend in aberration from normal to SMM and identified four TFs: *MAX*, *ZNF148*, *TCF4*, and *ZNF281*.

The gene module that *MAX* belongs to was determined to be condition-specific in normal samples. This means that the genes in the module were observed to be co-expressed in normal samples and less so in MGUS and SMM samples. This suggests that *MAX* is dysregulated in MGUS and SMM, which we observed to be true in the CNV data. MAX is known to complex with MYC to regulate transcription (Kato et al., 1992) and MYC is commonly known to be constitutively active in MM. The MAX–MYC relationship has been targeted in previous studies to inhibit c-MYC activity in MM cell lines (Holien et al., 2012). This association appears to conflict with our data, which shows the chromosomal region of *MAX* deleted in some MGUS and SMM samples and decreased gene expression in some SMM samples. An alternate explanation can be found in studies that show MYC can function independently of MAX in pheochromocytoma and small cell lung cancer (Ribon et al., 1994; Romero et al., 2014). MAX-independent expression of MYC in MM and its precursors requires further investigation; a recent abstract identified *MAX* as a tumor suppressor driver gene in MM (Garcia et al., 2017), which is a promising start.

*ZNF148* has been implicated in other MM studies (Magrangeas et al., 2003; Dong et al., 2015), but to our knowledge, none have directly associated this gene with MGUS or SMM. The associated chromosomal segment of *ZNF148* was progressively amplified from normal to MGUS and to SMM, corresponding with increased *ZNF148* gene expression. This suggests that this TF is involved as a driver in disease progression earlier than previously thought.

*TCF4* was differentially overexpressed in MGUS and SMM compared to normal. *TCF4* was not significantly amplified in MGUS, although this may be due to small sample size. We suggest that copy number amplification may play a part in *TCF4* dysregulation and may be involved in the initiation of MGUS but not SMM. This reasoning is due to the observation that the *TCF4* region is solely amplified in MGUS whereas there is a mix of amplified and deleted regions in SMM. This is consistent with our identification of *TCF4*’s gene module as MGUS-specific. Module enrichment and network analysis suggest Wnt signaling through *TCF4* contributes to *RUNX2* and *CCND1* overexpression. RUNX2 overexpression has been shown to be a driver of MM progression (Li et al., 2014; Trotter et al., 2015). *CCND1* overexpression has typically been observed to occur in MM precursors with chromosomal 11 and 14 translocations (Miura et al., 2003; Zhan et al., 2006). In gastric cancer, CCND1 has been shown to directly interact with TCF4 through the Wnt signaling pathway (Zheng et al., 2018), suggesting that other mechanisms of *CCND1* overexpression may also occur in MM.

*ZNF281* was increasingly amplified from MGUS to SMM patients. However, this is not considered statistically significant, possibly due to small sample size. Module enrichment results suggest transcriptional genes are more active in SMM, consistent with the fact that cancer cells require continued transcription in order to grow and proliferate. Increased transcription increases the chances of mutations in the DNA, which would activate tumor suppressor p53 and lead to cell cycle arrest or apoptosis in normal functioning cells. Cancer cells commonly have mutated *TP53* to avoid transcriptional control and apoptosis. However, *TP53* mutations are relatively rare in newly diagnosed MM patients (Chng et al., 2007b; Abdi et al., 2017). Our IPA network analysis suggests that TP53 may be regulated by CTNNB1. A previous study showed CTNNB1 suppressed TP53 in smooth muscle cells during artery formation (Riascos-Bernal et al., 2016). Something similar may be occurring in MM.

As previously observed by the original authors (López-Corral et al., 2012), the incidence of CNVs progressively increased from normal to MGUS and to SMM. Our analysis with Rawcopy identified similar regions of amplification and deletion from normal to MGUS and from MGUS to SMM. While not all the chromosomal regions were considered statistically different in the original study, it is visually striking how the frequency of chromosomal aberrations increase in patients from MGUS to SMM. The chromosomal regions of our identified TFs exhibited copy number changes. We suggest that these copy number alterations affect gene expression to an extent. The limitation is that we cannot offer direct evidence for this, therefore we suggest further exploration of this relationship in the laboratory.

There are other limitations to our study we should acknowledge. We filtered our gene lists down to 12,000–15,000 genes out of ∼22,000 and ∼54,000 microarray probes and identified TFs that showed consistent trends across groups. We may have removed or overlooked genes that could also play a part in myelomagenesis or progression. Although we inferred potential biological mechanisms of the four TFs from literature, the clinical significance of these genes remains to be investigated. Further research can be conducted to assess the pertinence of our TFs in addition to integrating other data modalities into more analyses. Despite these drawbacks, the biological details for these genes appear to have a relevant role in MM initiation and progression.

## Conclusion

In conclusion, we interrogated the role that TFs have in MM progression using a pipeline of GCN analysis, condition-specific gene module selection, TF enrichment analysis, and CNV analysis. We identified the TFs *MAX*, *ZNF148*, *TCF4*, and *ZNF281* from gene expression data and validated that their CNVs change from normal to MGUS and SMM. We examined the biological relevance of these TFs in MM and suggest further study of these genes in the laboratory.

## Author Contributions

KH and CY conceptualized the study. CY analyzed and interpreted the multiple myeloma data and was the major contributor in writing the manuscript. TJ, SX, and ZHu contributed to the design of the experiments and data interpretation. MA and XZ critically reviewed the manuscript. ZHa and KH gave research direction.

## Conflict of Interest Statement

The authors declare that the research was conducted in the absence of any commercial or financial relationships that could be construed as a potential conflict of interest.

## Footnotes

1 https://apps.medgen.iupui.edu/rsc/tsunami/

## Supplementary Material

FIGURE S1 Sample identity distograms of SMM samples produced by Rawcopy. **(A)** Distogram including GSM777173 that suggests this sample has some relatedness to other samples. **(B)** Distogram after removing GSM777173.

FIGURE S2 Gene-wise correlation heatmap of the two most highly similar modules in **(A)** lmQCM (*n* = 224) and **(B)** WGCNA (*n* = 393). The correlation coefficients are the absolute value of the Spearman correlation. The median correlation coefficient is higher in lmQCM (0.403) compared to WGCNA (0.344). SCC, Spearman correlation coefficient.

FIGURE S3 Gene-wise correlation heatmap of a normal-specific gene module. The genes in the module were identified by lmQCM in the normal samples. Gene-wise correlation coefficients are calculated from gene expression in each respective condition: **(A)** Normal, **(B)** MGUS, and **(C)** SMM. The correlation coefficients are the absolute value of the Spearman correlation. The genes are more correlated in normal samples and decrease in correlation in MGUS and SMM samples. The CCI values are 0.697, 0.226, and 0.252, respectively. SCC, Spearman correlation coefficient.

TABLE S1 GO BP enrichment results identified by enrichR. The most relevant enrichment terms are included along with the enrichment size and *p*-value associated with the corresponding dataset.
