# Supplementary figures and images for: Gene Co-expression Network and Copy Number Variation Analyses Identify Transcription Factors Associated With Multiple Myeloma Progression
Source: Front Genet. 2019 May 17;10:468. doi: 10.3389/fgene.2019.00468 (PMC6533571; doi:10.3389/fgene.2019.00468)

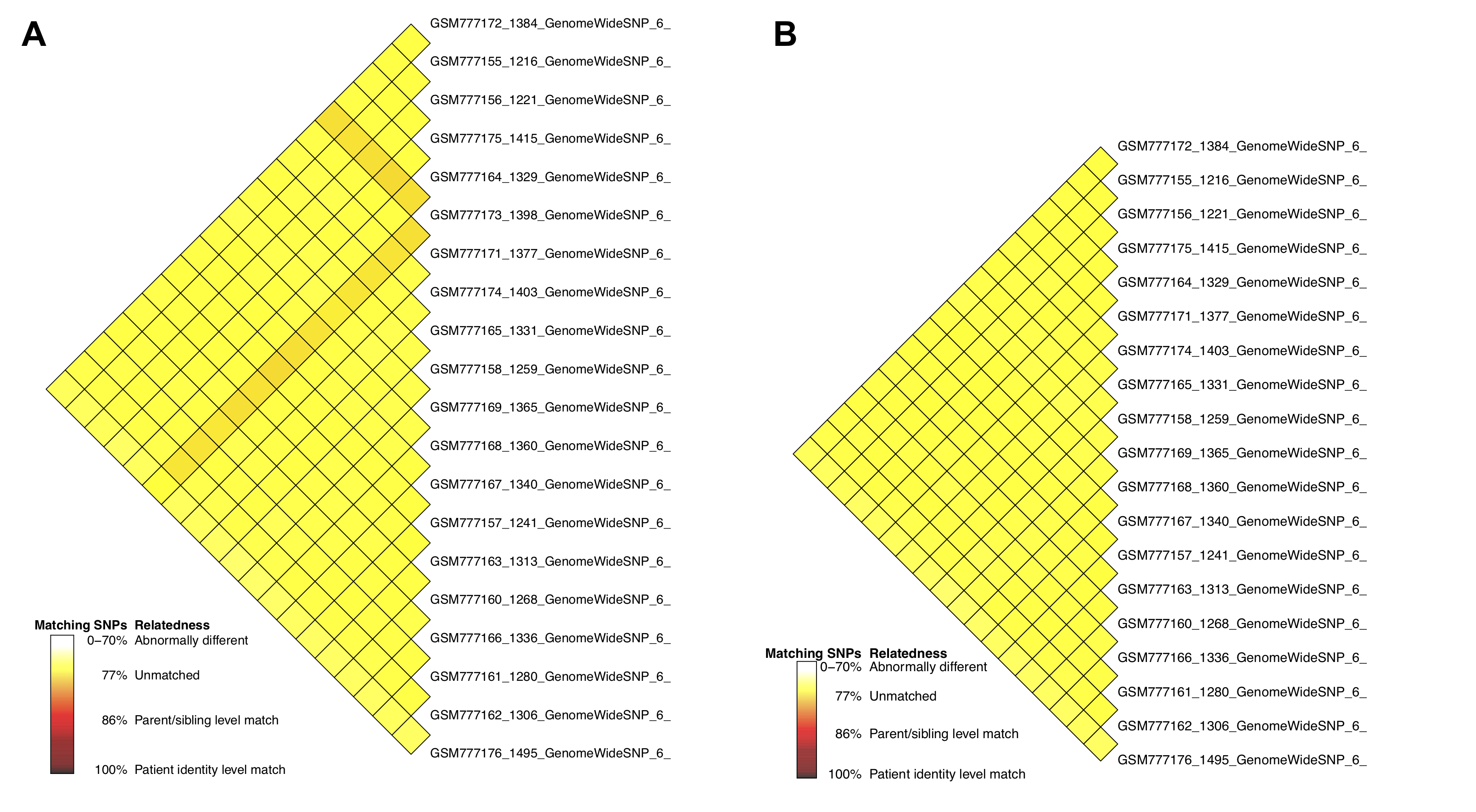

Supplement: FIGURE S1 — Sample identity distograms of SMM samples produced by Rawcopy. (A) Distogram including GSM777173 that suggests this sample has some relatedness to other samples. (B) Distogram after removing GSM777173. [file Image_1.TIFF]

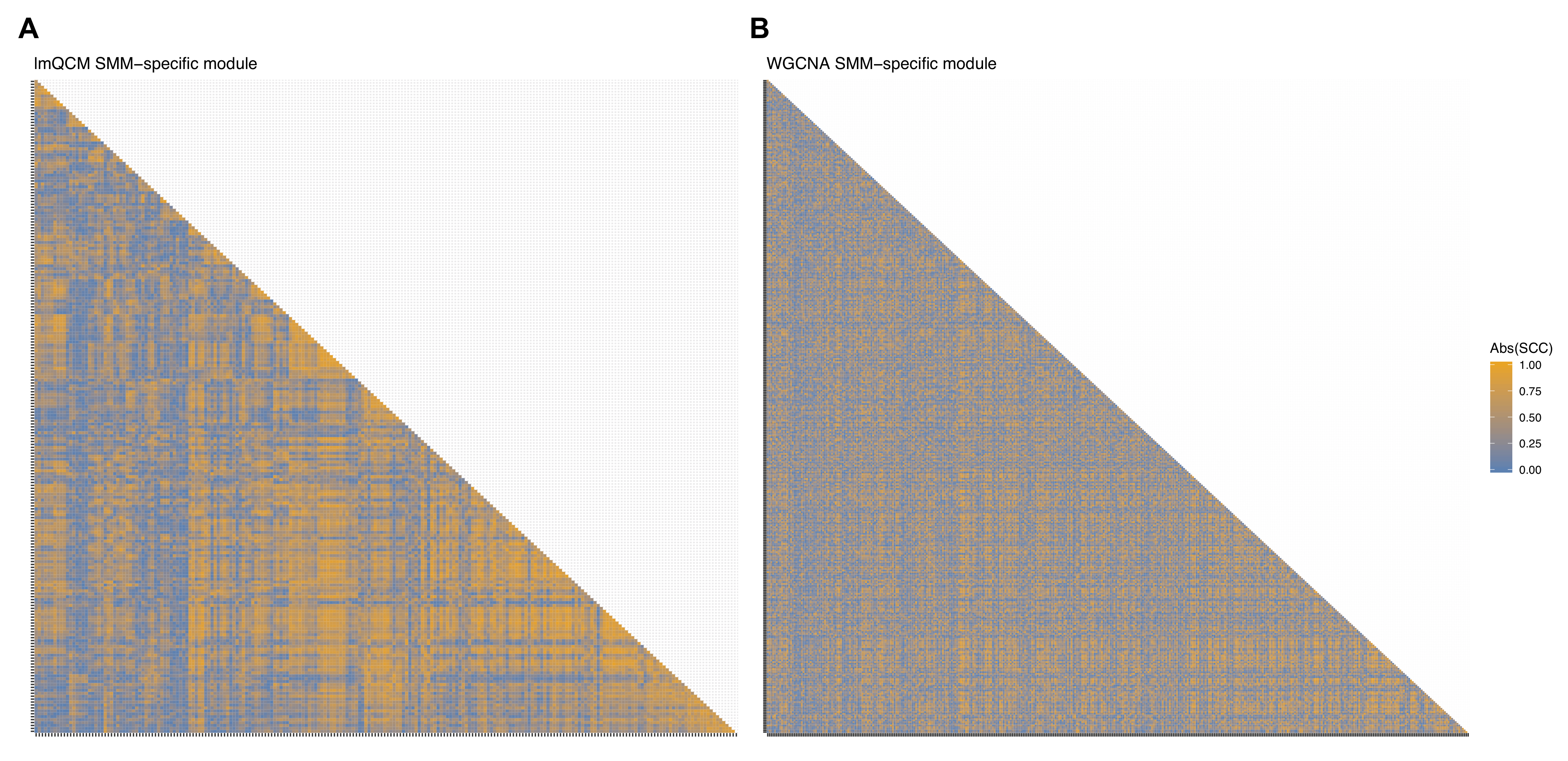

Supplement: FIGURE S2 — Gene-wise correlation heatmap of the two most highly similar modules in (A) lmQCM (n = 224) and (B) WGCNA (n = 393). The correlation coefficients are the absolute value of the Spearman correlation. The median correlation coefficient is higher in lmQCM (0.403) compared to WGCNA (0.344). SCC, Spearman correlation coefficient. [file Image_2.TIFF]

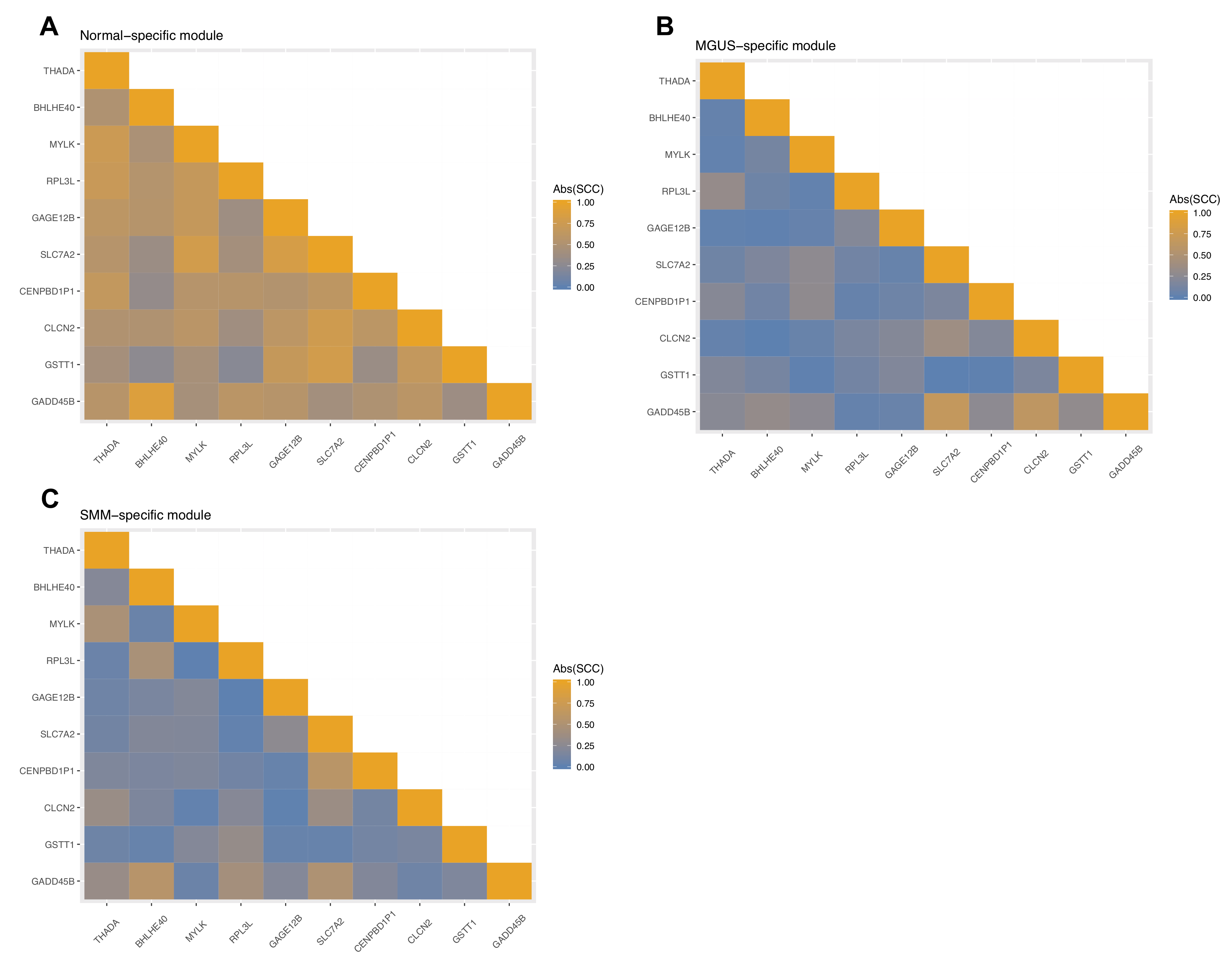

Supplement: FIGURE S3 — Gene-wise correlation heatmap of a normal-specific gene module. The genes in the module were identified by lmQCM in the normal samples. Gene-wise correlation coefficients are calculated from gene expression in each respective condition: (A) Normal, (B) MGUS, and (C) SMM. The correlation coefficients are the absolute value of the Spearman correlation. The genes are more correlated in normal samples and decrease in correlation in MGUS and SMM samples. The CCI values are 0.697, 0.226, and 0.252, respectively. SCC, Spearman correlation coefficient. [file Image_3.TIFF]
